# Study on the Irradiation Evolution and Radiation Resistance of PdTi Alloys

**DOI:** 10.3390/ma17184589

**Published:** 2024-09-19

**Authors:** Enbo Huo, Haochun Zhang, Yixin Liu

**Affiliations:** Institute of Nuclear Science and Technology, School of Energy Science and Engineering, Harbin Institute of Technology, Harbin 150006, China; 23b936198@stu.hit.edu.cn (E.H.); 24s002037@stu.hit.edu.cn (Y.L.)

**Keywords:** irradiation, defect clusters, PKA energy, elemental ratio, molecular dynamics

## Abstract

Medium-entropy alloys (MEAs) exhibit exceptional mechanical properties, thermal properties, and irradiation resistance, making them promising candidates for aerospace and nuclear applications. This study utilized molecular dynamics simulations to examine the defect behavior in PdTi alloys under various irradiation conditions. Simulations were performed using the Large-scale Atomic/Molecular Massively Parallel Simulator (LAMMPS) with the modified embedded-atom method (MEAM) potential to describe interatomic interactions. Various temperatures, primary knock-on atom (PKA) energies, and elemental ratios were tested to understand the formation and evolution of defects. The results show that compared to pure Pd, PdTi alloys with increased entropy exhibit significantly enhanced irradiation resistance at higher temperatures and PKA energies. This study explored the impact of different elemental ratios, including Pd, PdTi_1.5_, PdTi, and Pd_1.5_Ti. Findings indicate that increasing the Pd concentration enhances the alloy’s irradiation resistance, improving mobility and recombination rates of defect clusters. A one-to-one Pd-to-Ti ratio demonstrated optimal performance. Temperature analysis revealed that at 300 K and 600 K, PdTi alloys exhibit excellent irradiation resistance at a PKA energy of 30 keV. However, as the temperature rises to 900 K, the irradiation resistance decreases slightly, and at 1200 K, the performance is likely to decline further. This study offers some useful insights into the irradiation evolution and radiation resistance of PdTi medium-entropy alloys, which may help inform their potential applications in the nuclear field and contribute to the further development of MEAs in this area.

## 1. Introduction

Space nuclear power reactors and radioisotope thermoelectric generators (RTGs) play crucial roles in modern spacecraft. The high-temperature, high-radiation, and highly corrosive environment in space nuclear power reactors and radioisotope thermoelectric generators (RTGs) imposes greater demands on the materials’ ability to withstand extreme conditions, including heat, radiation, and corrosion. Therefore, it is crucial to develop novel reactor materials and conduct studies on their radiation damage resistance. Medium-entropy alloys (MEAs) have attracted widespread attention due to their excellent mechanical properties and radiation resistance, particularly alloy systems based on Al and fourth-period elements such as Fe, Co, Ni, Cr, Cu, Mn, Ti, and Pd [1,2,3,4,5]. As a medium-entropy alloy, PdTi is becoming an increasingly important subject of research. Compared to candidate cladding materials for space reactor cores, such as molybdenum–rhenium alloys, tungsten–rhenium alloys, TZM alloys, and T-111 alloys, the PdTi alloy is lighter, potentially offering a weight advantage for spacecraft applications. Furthermore, PdTi is a high-temperature shape-memory alloy, capable of recovering its original shape in high-temperature environments, making it a strong candidate for use in thermal protection and radiation shielding when constructing habitats or tents for extraterrestrial bases. It also shows potential as a candidate material for passive safety designs in space nuclear reactors. In addition, PdTi exhibits excellent corrosion resistance and hydrogen solubility, providing strong stability and resistance to hydrogen embrittlement in high-temperature hydrogen environments. This further suggests its suitability for hydrogen storage systems in space nuclear propulsion systems.

Researchers have conducted extensive experimental and theoretical studies on PdTi alloys. For example, as early as 1975, J. A. Horak and T. H. Blewitt focused on Pd and Ti, studying their annealing capabilities after thermal-neutron and fast-neutron irradiation. Their research demonstrated that these materials exhibited remarkable super-recovery phenomena following neutron irradiation [6]. In 1978, Rainer Kadel and Alarich Web investigated the solubility behavior of hydrogen in CuTi, CuTi_2_, PdTi_2_, and Cu_0.5_Pd_0.5_Ti_0.5_ alloys. Through experiments, they discovered that these titanium alloys exhibited varying solubility and diffusion characteristics in a hydrogen environment. The results indicated that PdTi alloys had a higher hydrogen solubility and significant hydrogen diffusion at elevated temperatures, suggesting potential advantages in stability and resistance to hydrogen embrittlement. These findings provide crucial insights for further understanding and optimizing the use of titanium alloys in hydrogen environments [7]. Huang et al. studied the structural energetics of PdTi and PtTi alloys using first-principles density functional theory (DFT), finding that they transition to a stable B198 ground state at low temperatures [8]. Mikusik et al. investigated PdTi-alloy formation on Ti surfaces using X-ray-excited photoelectron and Auger electron spectroscopy, discovering significant intermixing between Pd and Ti layers during annealing, forming a new PdTi_2_ phase [9]. Furthermore, atomic simulations have shown that PdTi high-temperature shape-memory alloys exhibit excellent shape-memory effects and shape-recovery capabilities in high-temperature environments [10]. Comparative studies of NiTi and PdTi alloys under cyclic loading have shown that PdTi alloys possess superior thermal stability and higher transformation temperatures, making them well-suited for high-temperature actuator applications [11]. Additionally, vacuum annealing studies have revealed that TiDy/Pd bilayer films exhibit different surface and bulk morphological changes compared to Ti/Pd films during annealing, promoting Ti diffusion into the Pd-rich top layer, making the formation of the PdTi_2_ phase more likely [12]. Electrochemical tests indicate that nanoporous PdTi alloys have significantly better catalytic activity and durability in oxygen-reduction reactions compared to traditional platinum-based catalysts, suitable for fuel-cell applications [13]. Not only PdTi but metal oxides such as TiO_2_, CeO_2_, Cu_2_O, and α-Fe_2_O_3_ also have widespread applications in catalysis [14,15], and the irradiation resistance of certain metal oxides has been extensively studied [16], with some being applied in space nuclear reactor materials. Therefore, further theoretical and simulation studies on the irradiation evolution and radiation damage resistance of PdTi alloys under high-temperature irradiation conditions are still essential for their application in high-temperature, high-radiation environments, such as space nuclear reactors and planetary surface bases.

Common methods used in irradiation damage studies include Monte Carlo (MC) methods, density functional theory (DFT), and molecular dynamics (MD) simulations. MC methods can simulate the formation and evolution of irradiation-induced defects, suitable for studying the accumulation of irradiation damage with high computational efficiency, but they lack detailed atomic-level structural information [17,18,19,20]. Density functional theory (DFT) [21,22,23,24,25,26,27,28,29,30,31] and first-principles methods are powerful tools in materials science, offering high precision and accurate predictions of electronic structures, chemical reactions, and mechanical properties. Their quantum mechanical foundation allows direct solutions to the Schrödinger equation, making them applicable to a wide range of materials, including metals, semiconductors, and insulators. However, these methods come with high computational costs that scale exponentially with system size. They are also generally suited for static or quasi-static problems, presenting challenges in simulating long-term dynamic processes. This paper aims to study the irradiation evolution process and radiation resistance of PdTi alloys. MD simulations can provide atomic-level dynamic behavior observations, directly simulating a more intuitive irradiation evolution process, revealing the generation, migration, and interaction of defects during irradiation while accommodating larger material systems. Therefore, MD is suitable for preliminary studies of larger material systems [32,33,34,35,36,37,38,39,40,41,42,43,44]. Thus, this study utilizes molecular dynamics simulations to investigate the irradiation evolution and radiation resistance of PdTi alloys, exploring the effects of entropy, elemental content, and temperature on the radiation resistance of PdTi alloys. Through these studies, we aim to provide theoretical support and guidance for the design and application of high-performance radiation-resistant materials in the future.

In this work, molecular dynamics (MD) simulations were performed using the Large-scale Atomic/Molecular Massively Parallel Simulator (LAMMPS) [45] to study the defect behavior in PdTi medium-entropy alloys (MEAs) during the displacement cascade and defect-formation and evolution stages under different PKA energies. Many studies have demonstrated that the MEAM potential offers significant advantages in studying the irradiation damage of PdTi alloys, as it can handle directional bonding and second-nearest-neighbor interactions, making it suitable for describing defect evolution and atomic rearrangement caused by irradiation. Compared to potentials such as EAM and Finnis–Sinclair, although more computationally demanding, the MEAM excels in precision and its ability to model complex alloy systems. Additionally, previous studies on PdTi alloys have also employed the MEAM potential, further validating its suitability and reliability in this field. In 2018, Won-Seok Ko, Jong Bae Jeon et al. demonstrated the reliability and applicability of the MEAM potential [10], developed by Lee, B.J. et al. using the 2NN MEAM framework, for modeling PdTi alloys by comparing it with experimental data and DFT calculations. Subsequently, B.J. Lee and G.U. Jeong further developed interatomic potentials for Pd-M (M = Al, Co, Cu, Fe, Mo, Ni, Ti) binary systems using the second-nearest-neighbor modified embedded-atom method to enable atomistic simulations for investigating atomic-scale structural evolution. These potentials successfully reproduced various fundamental properties of the alloys, aligning well with experimental data, first-principles calculations, and CALPHAD assessments [46]. In light of this, we utilized the latest PdTi potential developed by B.J. Lee and G.U. Jeong et al. [46] to conduct research on the irradiation evolution and radiation resistance of PdTi alloys.

## 2. Material and Method

The displacement cascade simulation boxes had dimensions of 40a × 40a × 40a, containing 2,256,000 atoms; 60a × 60a × 60a, containing 864,000 atoms; and 80a × 80a × 80a, containing 2,048,000 atoms. The PdTi Mesentropic alloy simulation unit was constructed by creating a random mixture of elements in a defined face-centered cubic (FCC) crystal containing equiatomic proportions of Pd and Ti elements. Based on the selected interatomic potential, the equilibrium lattice parameter (a) was set at 3.89 Å, as indicated by the potential file [46]. 

The displacement cascades were initiated by giving a Pd atom PKA energies ranging from 10 keV to 30 keV. In general, higher PKA energies result in more severe damage, increasing defect density, while lower energies produce fewer displacements and simpler defects. Zinkle and Kondo et al. [47] emphasized the crucial role of PKA energy in determining the extent and type of defects in materials. Higher PKA energies typically lead to more atomic displacements and more complex defect structures, while lower energies result in fewer and simpler defects. In many studies, 10 keV, 20 keV, and 30 keV have been widely used as PKA incident energy values to investigate irradiation-induced material evolution because they represent typical energy levels encountered in real irradiation scenarios. Choosing these energy values also ensures that our results are comparable with other research. The simulations used various ensembles to ensure accuracy and realism in the results. Initially, the system was relaxed using an NPT ensemble to equilibrate the pressure and temperature. This was followed by an NVT ensemble to maintain a constant temperature and volume during the cascade simulations. Finally, the actual cascade simulations were conducted under a constant NVE ensemble, with the ⟨100⟩ direction chosen as the PKA direction to avoid channeling effects. Before selecting the PKA incident direction, we initially explored different crystallographic orientations and even set the incident velocity direction of the PKA atoms to be arbitrary. By comparing the results of multiple simulation experiments, we found that these results were consistent and similar, with no significant impact on the following three key research areas: the effect of increasing entropy on the irradiation resistance of PdTi alloys, the effect of the Pd element ratio on the irradiation resistance of PdTi alloys, and the effect of temperature on the irradiation resistance of PdTi alloys. Therefore, this paper does not focus on the exploration or study of the incident direction. We ultimately chose the ⟨110⟩ direction for two main reasons: First, the results obtained in this direction were consistent and reliable, and second, selecting a specific direction helps readers better understand the experimental setup.

A variable MD time step (0.0001 fs to 0.001 fs) was used to speed up the simulation process, depending on the maximum displacement in the system (no more than 0.02 Å per atom per time step). To obtain representative statistics, five cascade simulations were repeated for each set of cascade energies. Each cascade simulation ran for 10 ps, which was sufficient for the cascade to anneal and return to a thermal equilibrium state. A thermostat was applied on the sides of the box to extract excess kinetic energy generated by the cascade by rescaling the velocities of atoms within a 5 Å thick layer, thereby minimizing the interaction of thermal waves with their periodic images. For the thermostat, we considered several methods, including the Berendsen thermostat, Nosé–Hoover thermostat, Langevin thermostat, and Velocity Rescaling thermostat. After comparison, we selected the Velocity Rescaling thermostat because it effectively handles excess kinetic energy in high-energy events (such as displacement cascades) while minimizing interference with the defect evolution within the system. This approach has also been validated and applied in related studies. For instance, Zepeda-Ruiz et al. employed a similar thermostat setup to control the system’s temperature by rescaling velocities, ensuring stability and accuracy in molecular dynamics simulations under extreme conditions [48]. Regarding the selection of boundary conditions and thermostat methods, we conducted thorough testing and analysis. Common boundary conditions include periodic boundary conditions, fixed boundary conditions, and free boundary conditions. We chose periodic boundary conditions because they simulate an infinite system, avoiding edge effects and providing a more physically meaningful simulation result. There is substantial literature supporting the effectiveness of periodic boundary conditions in ensuring the reliability of simulation results.

The formed interstitials and vacancies are identified using the Wigner–Seitz (W–S) cell method [49], where an empty W–S cell without any Pd or Ti atom is considered a vacancy, and two or more Pd (or Ti) atoms in the same W–S cell are considered to contain one or more interstitials. Based on previous studies, the second- or third-nearest-neighbor distance is used as the cutoff radius to define interstitial or vacancy clusters [50,51]. In this study, we treat three or more vacancy agglomerations and four or more interstitial agglomerations as clusters. Finally, the dislocation loops formed during the cascading processes are analyzed using the Open Visualization Tool (OVITO) [52] and the implemented dislocation extraction algorithm (DXA) [53].

**Figure 1:**
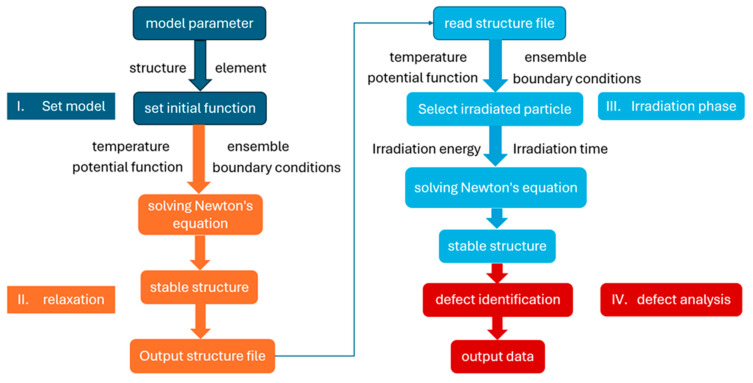
The molecular dynamics simulation process for radiation damage.

Figure 1 illustrates the molecular dynamics simulation process for radiation damage: During the initial model construction, develop the model based on research objectives and the scale of the simulation. In the relaxation phase, establish initial model conditions, including temperature, pressure, and boundary constraints. In the irradiation phase, execute simulation calculations through custom core programming. In the defect analysis phase, analyze and visualize the output from the simulation to assess defects.

In this study, palladium (Pd) atoms are selected as primary knock-on atoms (PKAs) for consistency, with their positions chosen randomly. The incident energies assigned are 10 KeV, 20 KeV, and 30 KeV. Due to the randomness of collisions, to ensure accurate results, this chapter selects 10 Pd atoms located in different positions for simulation calculations, and the results are analyzed and compared before taking the average. In molecular dynamics software, the energy of an atom is described by its velocity; therefore, it is necessary to convert the incident energy into velocity to be assigned to the PKA atom. The conversion formula is:

$$E=\frac{1}{2}mv^{2}\Rightarrow v=\sqrt{\frac{2E}{m}}$$

where *E* represents energy in joules (J), *m* represents the mass of the atom in kilograms (kg), and *v* is the velocity of the incident particle in meters per second (m/s). The related parameters are shown in Table 1.

## 3. Results

### 3.1. The Impact of Increasing Entropy on the Irradiation Resistance of PdTi Alloys

**Figure 2:**
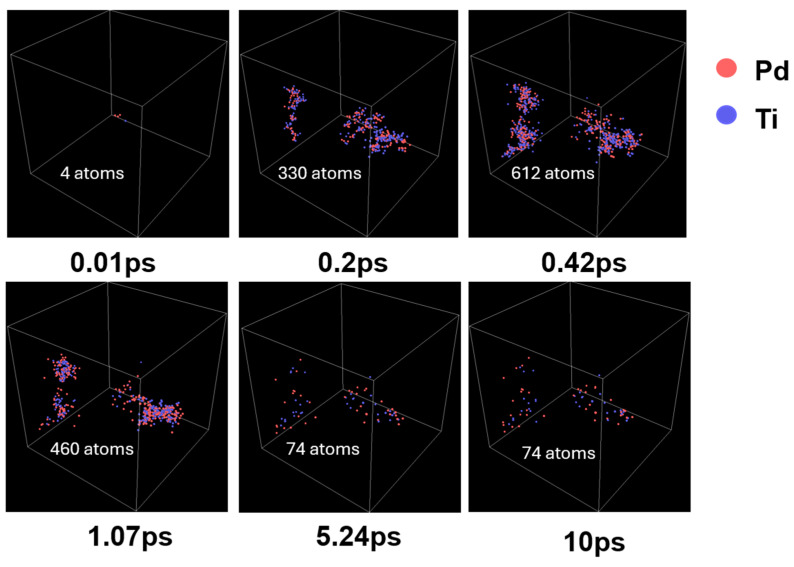
Evolution of irradiation damage in PdTi alloys at a temperature of 300 K with an incident particle energy of 10 keV.

During the incidence of a PKA atom, the cascade collision process can be observed. Figure 2 illustrates the evolution of irradiation damage in PdTi alloys at a temperature of 300 K with an incident particle energy of 10 keV. The figure shows that the PKA atom collides with atoms along its trajectory, transferring energy to these atoms. When the collision energy exceeds the displacement energy threshold, the atoms are displaced, creating displaced atoms. At the initial stage of the cascade collision, at 0.01 ps, a small number of point defects are generated. As the PKA atom continues to move and reaches 0.42 ps, the cascade collision peaks and the number of point defects reaches its maximum, although they are highly unstable. As the cascade collision progresses, these defects begin to recombine. By 1.07 ps, the number of point defects has significantly reduced. Over the following time, the point defects continue to recombine, eventually reaching a stable state around 5.24 ps. This process is consistent with the general behavior of irradiation damage in other alloys, but the irradiation damage in alloys is not entirely the same as that in other materials, such as metal oxides, with some exhibiting significant differences.

By comparing the irradiation evolution of CuO and ODS steels, it is evident that the irradiation evolution of PdTi alloys, CuO [54], and ODS steels [55] is different. Their distinct irradiation evolution also reflects the different mechanisms by which they respond to radiation. PdTi alloys show exceptional resistance to irradiation through a reduction in defect formation and enhanced defect recombination. During irradiation, primary knock-on atoms (PKAs) create point defects, but the unique composition of PdTi alloys limits the depth and extent of cascade collisions, preventing secondary defect clusters. CuO, on the other hand, exhibits a suppression of CuO formation in favor of Cu_2_O under irradiation, a process driven by radiation-induced defects that modify ion diffusion and oxidation behavior. ODS steels utilize dispersed nano-sized oxides to absorb radiation-induced defects, thereby enhancing their strength and stability in extreme environments such as nuclear reactors. However, maintaining the stability of these nano-oxides at high temperatures remains a challenge due to coarsening effects. These three materials emphasize the crucial role that defect formation and evolution play in determining their structural integrity and irradiation resistance.

**Figure 3:**
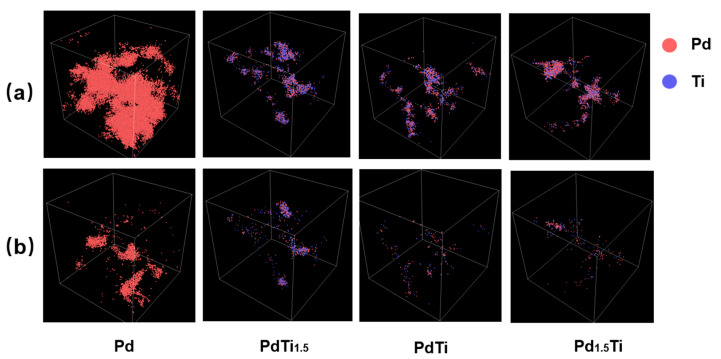
The distribution of the point defects at the peak of the cascade collisions (**a**) and the stabilized dis-tribution of the point defects after 10 ps of recombination (**b**) under a system temperature of 600 K and with a PKA incident energy of 20 keV for Pd, PdTi_1.5_, PdTi, and Pd_1.5_Ti are shown.

Figure 3 presents the distribution of the point defects at the peak of the cascade collisions and the stabilized distribution of the point defects after 10 ps of recombination under a system temperature of 600 K and with a PKA atom incident energy of 20 keV for Pd, PdTi_1.5_, PdTi, and Pd_1.5_Ti. Figure 3a depicts the scenario at the peak of the cascade collisions, and the bottom row (b) illustrates the scenario after 10 ps of irradiation. At the peak of the cascade collisions under 20 keV irradiation (Figure 3a), significant point defects can be observed. For the pure Pd, the point defects are widespread, with the cascade collisions penetrating the entire model and creating multiple secondary cascade clusters, indicating minimal obstruction. In the PdTi_1.5_ alloy, the depth and size of point-defect distribution are reduced, but secondary cascade clusters still exist, indicating some degree of obstruction. In the PdTi alloy, the depth and size of the point defects are further reduced, and no secondary cascade clusters are observed, suggesting significant obstruction of the cascade collision.

The definition of high-entropy alloys stems from their high mixing entropy. In thermodynamic processes, entropy measures the degree of disorder in a system. The increase in system entropy (*S*) is synchronous with the increase in the mixing degree (Ω), meaning the greater the mixing, the larger the entropy value. According to the statistical thermodynamic hypothesis by Boltzmann, the system entropy is related to the system mixing degree by the following formula: *S* = *k*lnΩ. In this equation, *k* is the Boltzmann constant, 1.38054 × 10^23^ J/K. For a pure alloy system, the total entropy includes the mixing entropy (also called configurational entropy, *S*_conf_), magnetic entropy (*S*_mag_), vibrational entropy (*S*_v_), and electronic entropy (*S*_e_). In the changes in different combined entropies, the mixing entropy contributes the most compared to the others. Therefore, changes in the entropy of the alloy system are often represented by the mixing entropy Δ*S*_*conf*_ in solid-solution or liquid-solution alloys.

$$S=k\ln\Omega =-R\sum_{i==1}^{n}X_{i}\ln X_{i}$$

where *R* is the molar gas constant, *R* = 8.314 J/(K⋅mol), and *X_i_* is the atomic fraction of element *i* in the alloy. The first term in the formula represents the number of atoms; when the atomic fractions of the components in the alloy are equal, the system achieves the maximum mixing entropy. It is known that as the number of components in the alloy system continues to increase, the corresponding system’s mixing entropy also increases. The components of the alloy can be any metallic elements. When *n* = 2 and 5, Δ*S*_*conf*_ is 0.69 R and 1.61 R, respectively. When the number of components increases beyond five, the growth rate of the system’s mixing entropy gradually decreases. The most direct manifestation is that the slope gradually flattens. When *n* exceeds 10, the rate of increase in the mixing entropy of the system slows down significantly. This suggests that when *n* reaches a certain value, the influence of increasing *n* on the mixing entropy becomes quite limited. Enhancing the mixing entropy cannot be achieved merely by increasing the number of main components.

The calculated entropy values for pure Pd, PdTi_1.5_, PdTi, and Pd_1.5_Ti are 0, 0.673 R, 0.693 R, and 0.673 R, respectively. From the irradiation damage graphs of the first three alloys (Pd, PdTi_1.5_, and PdTi), it is evident that as the entropy increases and the Pd content rises, the alloys’ irradiation resistance significantly improves, with PdTi showing a marked enhancement in performance. However, the Pd_1.5_Ti alloy displays a similar depth and size of point-defect distribution compared to PdTi, indicating that the irradiation resistance does not improve further with increased Pd content, potentially due to the reduction in entropy. 

**Figure 4:**
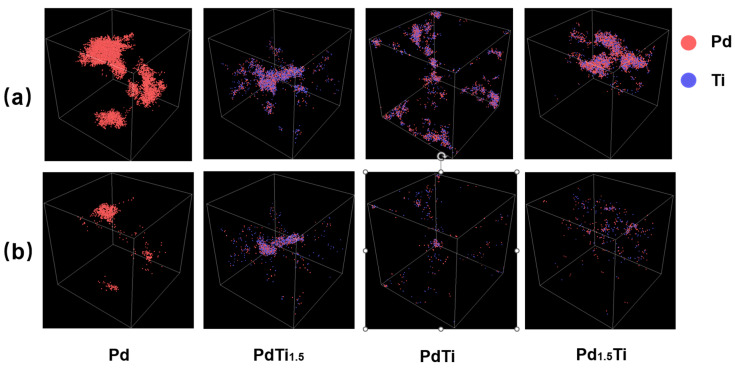
The distribution of the point defects at the peak of the cascade collisions (**a**) and the stabilized distribution of the point defects after 10 ps of recombination (**b**) under a system temperature of 600 K and with a PKA incident energy of 30 keV for Pd, PdTi_1.5_, PdTi, and Pd_1.5_Ti are shown.

Figure 4 presents the distribution of the point defects at the peak of the cascade collisions and the stabilized distribution of the point defects after 10 ps of recombination under a system temperature of 600 K and with a PKA atom incident energy of 30 keV for Pd, PdTi_1.5_, PdTi, and Pd_1.5_Ti. Figure 3a depicts the scenario at the peak of the cascade collisions, and the bottom row (b) illustrates the scenario after 10 ps of irradiation. At the peak of the cascade collisions under 30 keV irradiation (Figure 4a), the point defects increase significantly across all alloys compared to the 20 keV scenario. For pure Pd, the point defects are even more widespread, with deeper penetration and multiple secondary cascade clusters, indicating continued minimal obstruction, which is similar to the trend observed in the results for Ni in the referenced literature [56,57]. In the PdTi_1.5_ alloy, the depth and size of the point-defect distribution are still reduced, but secondary cascade clusters persist. In the PdTi alloy, the point defects are further reduced, with no secondary cascade clusters, indicating significant obstruction of the cascade collision. The Pd_1.5_Ti alloy shows a similar pattern to PdTi, suggesting that increasing Pd content does not improve irradiation resistance further, likely due to the reduced entropy.

After 10 ps of irradiation under 30 keV (Figure 4b), the distribution of the point defects stabilizes but remains more pronounced than in the 20 keV scenario [57]. In pure Pd, the defects remain extensive. The PdTi_1.5_ alloy continues to exhibit some secondary cascade clusters, but when the incident PKA atom energy is increased from 20 keV to 30 keV, the PdTi_1.5_ alloy does not show reduced defect depth and size compared to pure Pd, nor does it demonstrate an advantage in defect recovery capability. The PdTi alloy maintains significantly reduced defects, with no secondary clusters, indicating strong obstruction. The defect distribution of Pd_1.5_Ti appears to be smaller than that of PdTi. This improvement might be due to the increased Pd content, whose advantage becomes more significant than the entropy increase when the incident PKA atom energy is raised from 20 keV to 30 keV.

Although this study focuses on a binary PdTi alloy, which differs from high-entropy alloys, medium-entropy alloys (MEAs) still exhibit significant effects that can help explain the enhanced irradiation resistance observed in PdTi. The medium-entropy effect refers to the relatively high mixing entropy of medium-entropy alloys, which helps form a stable solid solution, thereby enhancing the thermal stability of the alloy under irradiation. Additionally, the difference in atomic radii between Pd and Ti leads to a lattice distortion, increasing the resistance to dislocation motion and defect formation, which helps mitigate the expansion of radiation-induced defects. In PdTi alloys, these effects collectively explain the alloy’s superior irradiation resistance. First, the medium-entropy effect contributes to the formation of a stable solid-solution structure, and compared to pure Pd, the addition of Ti enhances the thermodynamic stability of the alloy under irradiation, thereby reducing structural damage caused by phase transformations. Second, the lattice distortion effect increases lattice strain in PdTi alloys, further strengthening the resistance to dislocation motion and point-defect formation, ultimately reducing the expansion of radiation-induced defects. The synergy of these effects makes PdTi alloys highly effective in resisting irradiation damage.

### 3.2. The Impact of the Pd Element Ratio on the Irradiation Resistance of PdTi Alloys

**Figure 5:**
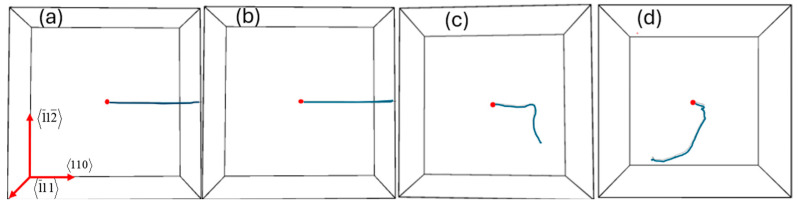
Trajectories of PKA in (**a**) Pd, (**b**) PdTi_1.5_, (**c**) PdTi, and (**d**) Pd_1.5_Ti alloys. The red arrows indicate the crystallographic directions, while the blue lines depict the motion trajectories of the PKA in each alloy system. The red dot at the center of each subfigure (**a**–**d**) represents the starting point of the PKA’s motion.

Figure 5 shows the trajectories of primary knock-on atoms (PKAs) in different PdTi alloys at 600 K under a PKA atom incident energy of 20 keV. The images depict the movement of the PKA atoms in the four different alloy compositions: (a) PdTi, (b) PdTi_1.5_, (c) PdTi, and (d) Pd_1.5_Ti. In the PdTi alloy (a), the trajectory of the PKA atom appears relatively straight and covers a considerable distance. This indicates that the PKA atom moves through the lattice with minimal deflection, suggesting that the lattice provides less resistance to the PKA atom’s motion. This could imply relatively fewer interactions with surrounding atoms, leading to fewer collisions and, thus, less energy dissipation. In the PdTi_1.5_ alloy (b), the PKA atom’s trajectory is also relatively straight and similar to that in pure Pd. This suggests that the PKA atom encounters a similar level of resistance in PdTi_1.5_ as in pure Pd, resulting in comparable energy dissipation. The trajectory shown for PdTi (c) indicates more deflection and the shortest path among the alloys. This suggests a higher degree of interaction between the PKA atom and the surrounding lattice atoms, leading to more collisions and energy dissipation. The increased deflection implies that the PKA atom loses energy more quickly, resulting in a shorter overall trajectory. The PKA atom in Pd_1.5_Ti (d) exhibits a curved trajectory with a short path. This indicates a high level of interaction with the lattice atoms, leading to multiple collisions and substantial energy dissipation. Although the path is slightly longer than that in PdTi (c), it still shows significant energy dissipation. Overall, the trajectories of the PKA atoms in pure Pd and PdTi_1.5_ alloys are similar, indicating comparable levels of energy dissipation and obstruction. The PdTi alloy shows the shortest and most deflected trajectory, indicating the highest energy dissipation and strongest interaction with the lattice [56,58]. The Pd_1.5_Ti alloy’s trajectory is similar to PdTi, showing high interaction and significant energy dissipation.

Regarding energy dissipation quantification, we used molecular dynamics (MD) simulations to track the energy loss of primary knock-on atoms (PKAs) as they interacted with the lattice. The dissipation was monitored by measuring the decrease in kinetic energy over time and its conversion into lattice vibrations and atomic displacements. To assess the trajectory deviations, key parameters included the PKA’s initial and final positions, the deflection angle, and the number of collisions with lattice atoms. We visualized the trajectory using tools such as OVITO to observe the PKA’s motion. The rate of energy dissipation was calculated by monitoring the PKA’s kinetic energy at each time step and summing the energy transferred to surrounding atoms. This allowed us to correlate the energy loss with the trajectory deviations and collision frequency.

Since point defects such as interstitial atoms and vacancies invariably form Frenkel defects, a detailed analysis of the temporal evolution in point defect numbers was conducted by statistically tracking the Frenkel pair count over time. For successful recombination, the standard was based on observing a reduction in interstitial and vacancy atoms from the data. Specifically, the Wigner–Seitz defect analysis in OVITO was used to track how displaced atoms rejoined their original lattice positions. When a displaced atom returned within a cutoff distance (√2 times the lattice constant) of its original position, we considered this a successful recombination. Regarding long-term migration effects, the driving forces for long-term migration are primarily atomic vibrations and the presence of local stress fields within the lattice, while temperature could also play a possible role. Although recombination can still occur during long-term migration, the likelihood decreases as defects become more widely dispersed and the potential for mutual interaction diminishes. In our simulations, we observed some degree of recombination during these extended timescales, but it was significantly less than during the initial collision stages.

**Figure 6:**
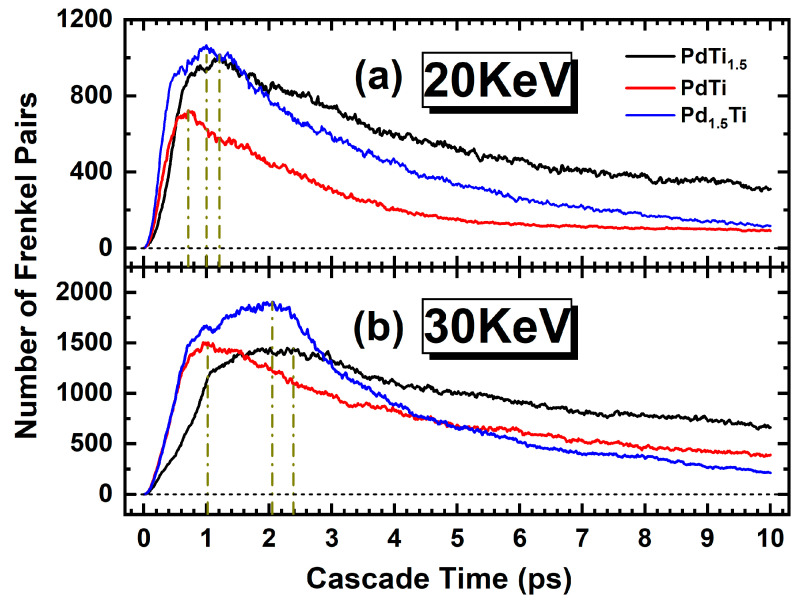
The number of Frenkel pairs produced as a function of simulation time in single crystals of Pd, PdTi_1.5_, PdTi, and Pd_1.5_Ti at a temperature of 600 K with PKA energies of (**a**) 20 keV and (**b**) 30 keV. The black, red, and blue lines correspond to the results for PdTi_1.5_, PdTi, and Pd_1.5_Ti alloys, respectively.

As depicted in Figure 6, the curves represent the Frenkel pair count as a function of time, averaged across 10 datasets. To enable a clear and unambiguous observation of the temporal evolution of the Frenkel pairs, error bars are omitted. Despite this, each calculation inherently exhibits minor fluctuations, which remain within 5% of the nominal value. A comprehensive analysis reveals that the trend in the Frenkel pair count closely mirrors the trends observed in irradiation simulation results for other alloys [59]. This congruence likely arises from the irradiation-induced alterations in crystal structure and atomic interactions. Irradiation triggers processes such as atomic displacement, defect formation, and migration, which are ubiquitous across various alloy systems. Consequently, the observed trends in the Frenkel pair count from irradiation simulations may possess universal applicability to diverse alloy systems. Initially, the Frenkel pair count in the three metals reaches a zenith within an exceedingly brief period. The temporal evolution of the Frenkel pair count can be delineated into four distinct stages: During the primary knock-on stage (0–0.1 ps), the Frenkel pair count escalates rapidly, attaining the thermal spike stage (0.1–3 ps) for a remarkably short duration, where the Frenkel pair count peaks. Subsequently, the cascade collision transitions into the ballistic collision residual stage, wherein the Frenkel pairs further evolve and recombine. The duration of the ballistic collision residual stage varies substantially depending on the alloy. Finally, in the defect migration stage, the remaining Frenkel pairs from the preceding stage undergo further recombination and annihilation, resulting in a continued diminution in defect numbers. The termination time of the defect migration stage also varies significantly based on the alloy. The evolution of the Frenkel pair count in these metals traverses through the primary knock-on stage, thermal spike stage, ballistic collision residual stage, and defect migration stage. These stages encapsulate the processes of defect formation, accumulation, recombination, and migration at the atomic scale.

In Figure 6a, corresponding to an initial energy of 20 keV, the red curve represents the PdTi alloy, the black curve represents the PdTi_1.5_ alloy, and the blue curve represents the Pd_1.5_Ti alloy. It is evident that the number of Frenkel pairs increases rapidly at the onset, reaching a peak and subsequently decreasing gradually over time. In Figure 6b, where the initial energy is 30 keV, the red curve corresponds to the PdTi alloy, the black curve to the PdTi_1.5_ alloy, and the blue curve to the Pd_1.5_Ti alloy. The behavior is similar to the 20 keV case, with the number of Frenkel pairs initially rising sharply, peaking, and then diminishing over time. However, both the peak number of Frenkel pairs and the overall scale are notably higher in the 30 keV case compared to the 20 keV scenario.

In both panels, the evolution of Frenkel pairs indicates that the PdTi_1.5_ alloy consistently exhibits the highest number of defects, followed by the Pd_1.5_Ti alloy, with the PdTi alloy showing the lowest number of defects. Additionally, a noticeable shift in the peak position is observed, suggesting that the peak time varies with the alloy composition and initial energy, thereby underscoring the significant influence of both factors on the defect dynamics in these alloys [59].

**Figure 7:**
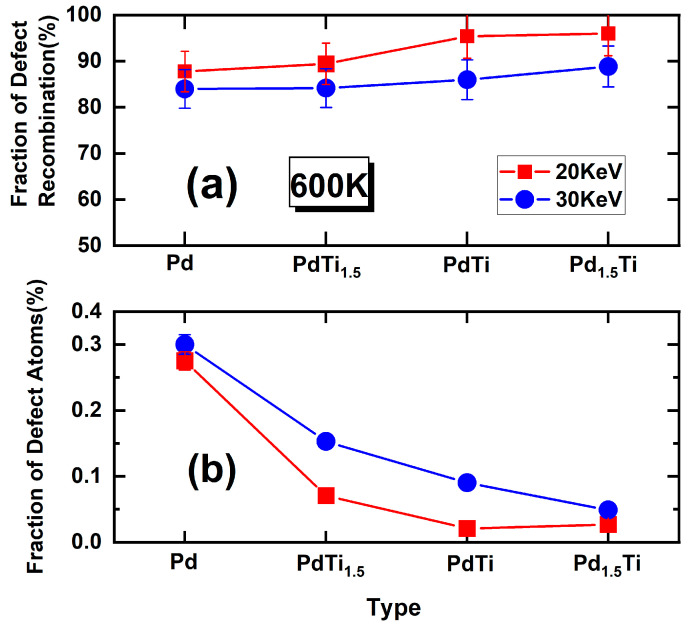
(**a**) Composite rate curves of four alloys and (**b**) the percentage of defect atoms relative to the total number of atoms in the system. The red and blue lines correspond to the results for PKA energies of 20 keV and 30 keV, respectively.

The defect recombination rate is a crucial metric for assessing irradiation resistance. It is defined as the percentage of interstitial atoms returning to lattice positions relative to the total number of defects. According to the Kinchin–Pease (K–P) theoretical model, the number of defects in the steady state can be calculated. Figure 7a shows the recombination rate curves for pure Pd and three PdTi alloys after 10 ps of irradiation at 600 K. From the figure, it can be observed that at a PKA energy of 20 keV, the recombination rates of Pd and PdTi_1.5_ are below 90%, while those of PdTi and Pd_1.5_Ti exceed 90%. As the incident energy increases to 30 keV, the defect recombination rates of the four metals slightly decrease. When the incident energy is 20 keV, the recombination rates of Pd, PdTi_1.5_, and PdTi increase sequentially, with PdTi and Pd_1.5_Ti showing the highest recombination rates, which are almost identical. At an incident energy of 30 keV, the recombination rates of Pd and PdTi_1.5_ are similar. The recombination rate of PdTi is higher than that of Pd and PdTi_1.5_ but slightly lower than that of Pd_1.5_Ti. This suggests that as the incident PKA energy increases to 30 keV, the Pd content in the alloys enhances their irradiation resistance, and this factor outweighs the advantage of the higher entropy in PdTi.

From Figure 7b, it is evident that as the incident PKA energy increases, the destructive impact on the alloy structure intensifies, resulting in a higher proportion of defect atoms in the system. Regardless of whether the incident PKA energy is 20 keV or 30 keV, the proportion of defect atoms in the Pd, PdTi_1.5_, and PdTi alloys decreases sequentially. This indicates that the increase in entropy positively contributes to the irradiation resistance of the materials. At 20 keV, the PdTi alloy exhibits the smallest proportion of defect atoms. When the incident PKA energy increases to 30 keV, the proportion of defect atoms in Pd, PdTi_1.5_, PdTi, and Pd_1.5_Ti alloys decreases with the increasing Pd content, with Pd_1.5_Ti showing a lower proportion of defect atoms than PdTi. This suggests that at an incident PKA energy of 30 keV, increasing the Pd content in the alloy plays a more significant role in enhancing irradiation resistance.

During irradiation, defects migrate and coalesce to form stable clusters, including interstitial atom clusters and vacancy clusters. The size and quantity of these defect clusters are critical indicators of a material’s irradiation resistance. Larger defect clusters can induce local stress concentrations and lattice distortions, rendering the material more prone to fracture, crack propagation, and other failure mechanisms, thereby accelerating its degradation. Specifically, the aggregation of large vacancy clusters results in voids that occupy the crystal lattice, reducing material density and causing macroscopic swelling behavior [60,61].

In this study, the cluster analysis method in OVITO was utilized to assess the size of defect clusters by setting an appropriate cutoff distance. The cutoff distance was chosen as √2a (where a represents the lattice constant), thus being set to 5.5 Å. This implies that during the cluster analysis, OVITO identifies particles within a distance of 5.5 Å as connected, thereby classifying them into clusters accordingly. The size of the defect clusters in this study is determined by the number of interstitial atoms and vacancies within each cluster. The aggregation of two or more interstitial atoms or vacancy defects is defined as a cluster [62].

**Figure 8:**
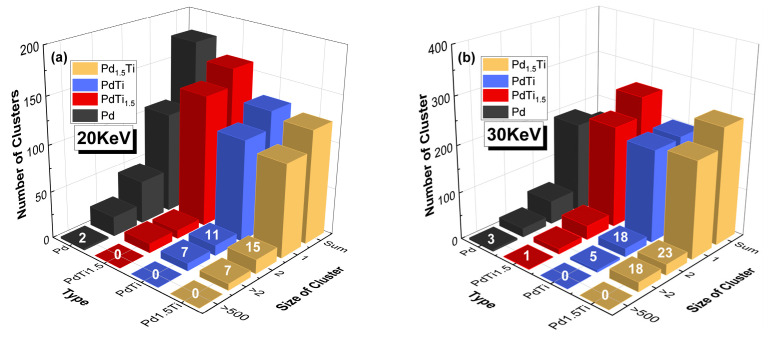
Defect cluster size distribution for Pd, PdTi_1.5_, PdTi, and Pd_1.5_Ti alloys at 600 K with incident PKA energies of (**a**) 20 keV and (**b**) 30 keV. The numbers above the bars indicate the exact number of defect clusters for each alloy and cluster size.

Figure 8 shows the defect cluster size distribution for Pd, PdTi_1.5_, PdTi, and Pd_1.5_Ti alloys at 600 K with incident PKA energies of (a) 20 keV and (b) 30 keV. At an incident energy of 20 keV, Pd and PdTi_1.5_ alloys, in particular, exhibit a higher number of clusters across different size ranges, indicating significant defect aggregation. The cluster quantities for PdTi and Pd_1.5_Ti are similar, suggesting comparable defect clustering behavior at this energy level. When the incident energy increases to 30 keV, Pd and PdTi_1.5_ show an increase in both defect cluster size and quantity. Notably, PdTi has the smallest cluster sizes among all alloys. Although Pd_1.5_Ti has a higher number of atoms not equal to 1 compared to PdTi at 30 keV, its overall cluster size is larger than that of PdTi, which is an interesting phenomenon. As the incident PKA energy increases from 20 keV to 30 keV, the overall number of clusters with sizes of 1, 2, greater than 2 but less than 500, and greater than 500 increases. After 10 ps of irradiation, neither PdTi nor Pd_1.5_Ti shows clusters larger than 500 at 20 keV or 30 keV. As the PKA energy increases from 20 keV to 30 keV, PdTi forms smaller defect clusters, demonstrating the best irradiation resistance.

**Figure 9:**
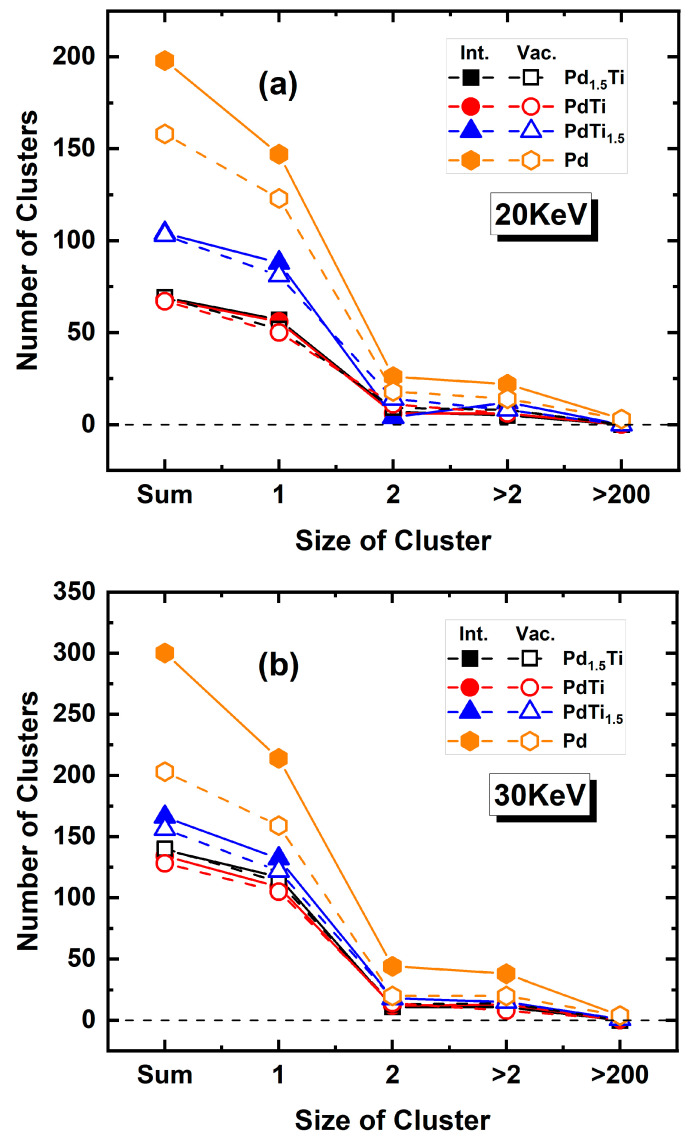
Comparison of the number of interstitial atoms (occupancy = 2) and vacancy atoms (occupancy = 0) in different cluster sizes for Pd, PdTi_1.5_, PdTi, and Pd_1.5_Ti alloys at 600 K with incident PKA energies of (**a**) 20 keV and (**b**) 30 keV. Hollow symbols and solid lines represent the number of interstitial atoms of the corresponding size, while filled symbols and dashed lines represent the number of vacancy atoms of the corresponding size.

Figure 9 presents a comparison of the number of interstitial atoms (occupancy = 2) and vacancy atoms (occupancy = 0) in different cluster sizes for Pd, PdTi_1.5_, PdTi, and Pd_1.5_Ti alloys at 600 K with incident PKA energies of (a) 20 keV and (b) 30 keV. It is observed that the defect clusters in these alloys predominantly exist in the form of single defects, with only a small number of clusters forming with sizes of 2, 3–500, and greater than 500. The number of clusters with sizes of 2, 3–500, and greater than 500 increases with the incident energy. PdTi exhibits the least formation of larger clusters. Furthermore, interstitial atoms are more likely to aggregate and form clusters in the size range of 3–10, whereas vacancy defects are less likely to form clusters. Studies indicate that vacancies are less mobile than interstitials because the atoms around a vacancy in a crystal form a binding effect that restricts the movement of the vacancy. As the number of defects increases, their mobility decreases, making vacancy clusters more prone to being confined by surrounding atoms and thus forming smaller clusters. In contrast, the atoms around interstitial defects are more loosely arranged, allowing interstitials to move more easily. Therefore, individual vacancies are more likely to form smaller clusters.

**Figure 10:**
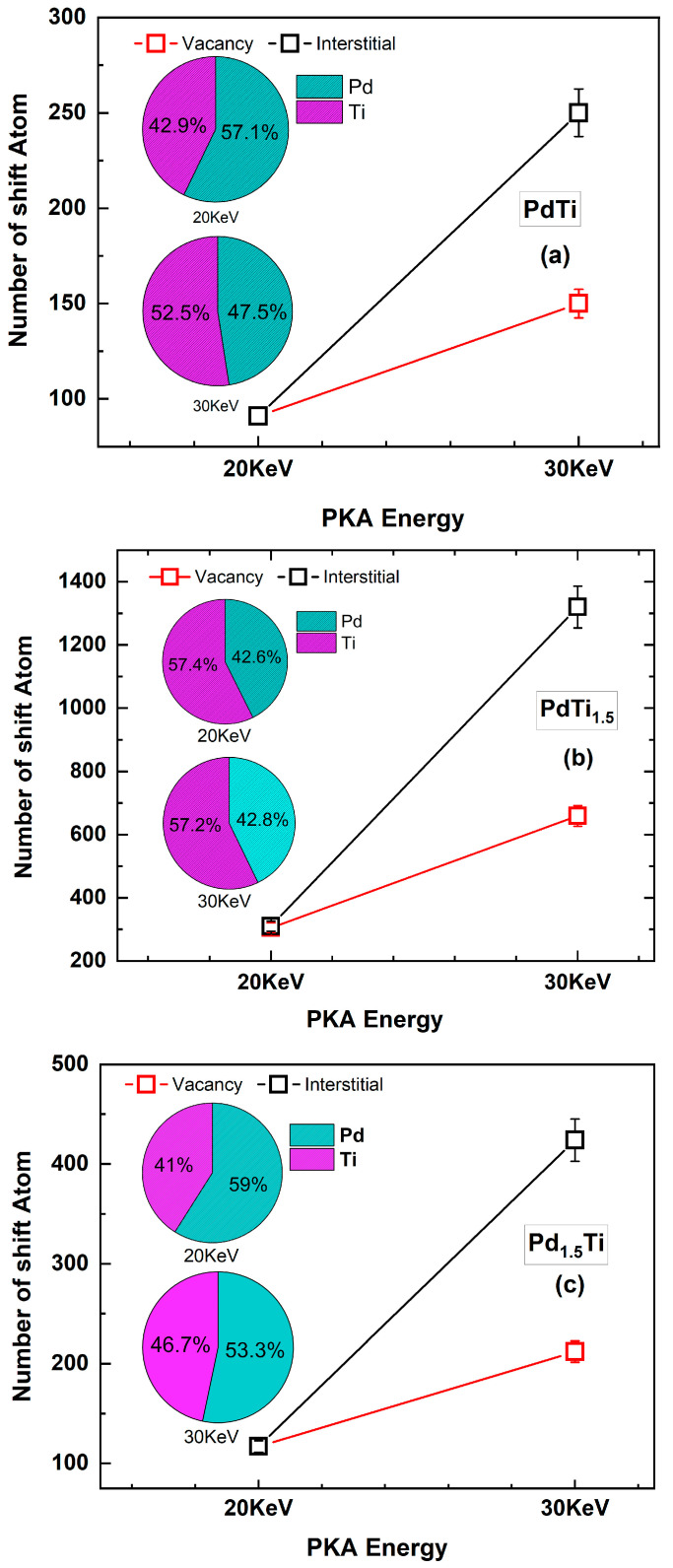
Variation of defect cluster atom numbers with PKA energy. The pie charts approximately show the proportion of Pd and Ti in interstitial atom clusters at different PKA energies for (**a**) PdTi, (**b**) PdTi_1.5_, and (**c**) Pd_1.5_Ti.

Figure 10 describes the variation in the number of defect cluster atoms with PKA energy for (a) PdTi, (b) PdTi_1.5_, and (c) Pd_1.5_Ti alloys after 10 ps of irradiation. According to Figure 10, as the PKA energy increases from 20 keV to 30 keV, the number of interstitial atoms and vacancies in the defects also increases. The interstitial atoms increase the fastest and constitute the main part of the defect clusters, which is consistent with the conclusions in Figure 9. Therefore, we explored the elemental composition of interstitial atoms in these clusters. From the pie charts in Figure 10, it is evident that as the PKA energy increases from 20 keV to 30 keV, the Ti atom content in the interstitial atoms of defect clusters in (a) PdTi and (c) Pd_1.5_Ti alloys increases to some extent. During this process, the Ti atom content in the PdTi_1.5_ alloy does not increase; however, from the pie chart in Figure 10b, it can be observed that the Ti atom content in the PdTi_1.5_ alloy is already as high as 57.4%. This further indicates that as the PKA energy increases, Ti atoms, compared to Pd atoms, can increase the number of interstitial atoms in defect clusters in PdTi alloys, making them an important factor in increasing the number of atoms.

Therefore, we found that when irradiating the PdTi alloy at PKA energies of 20 keV and below, the irradiation resistance of the alloy gradually enhances with the increase in entropy. As the PKA energy increases to 30 keV, the Pd component in the PdTi alloy demonstrates its stability, while the Ti atoms in the PdTi alloy are more likely to be irradiated into interstitial atoms.

### 3.3. The Impact of Temperature on the Irradiation Resistance of PdTi Alloys

**Figure 11:**
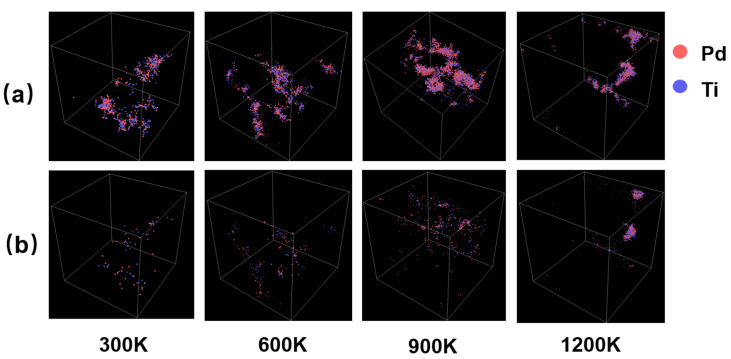
(**a**) The distribution of point defects at the peak of cascade collisions for PdTi at different temperatures (300 K, 600 K, 900 K, and 1200 K) with a PKA atom incident energy of 20 keV. (**b**) The stabilized distribution of point defects after 10 ps of evolution under the same conditions. Red represents Pd atoms, and blue represents Ti atoms.

Figure 11 illustrates the distribution of the point defects in PdTi at different temperatures (300 K, 600 K, 900 K, and 1200 K) with an incident PKA energy of 20 keV, both at the peak of the cascade collisions and after 10 ps of recombination. At 300 K, the depth and size of the point-defect distribution generated by cascade collisions are relatively small, with defects clustered near the PKA atom and no secondary cascade clusters observed. After 10 ps of recombination, the stable point-defect distribution significantly decreases in depth and size, leaving only a few atoms that did not return to their original positions. As the temperature increases to 600 K, the distribution of the point defects generated by cascade collisions shows little change compared to 300 K. However, after 10 ps of recombination, the number of stable point defect atoms significantly decreases, indicating an effective recombination process. At 900 K, the number of displaced atoms due to cascade collisions increases compared to 600 K, but after 10 ps of recombination, the number of atoms occupying non-regular lattice sites greatly reduces, with no large clusters observed. When the temperature rises to 1200 K, the depth and size of the point-defect distribution generated by cascade collisions are similar to those at 900 K. However, after 10 ps of recombination, despite a large number of atoms recombining, two relatively distinct clusters are still observed.

**Figure 12:**
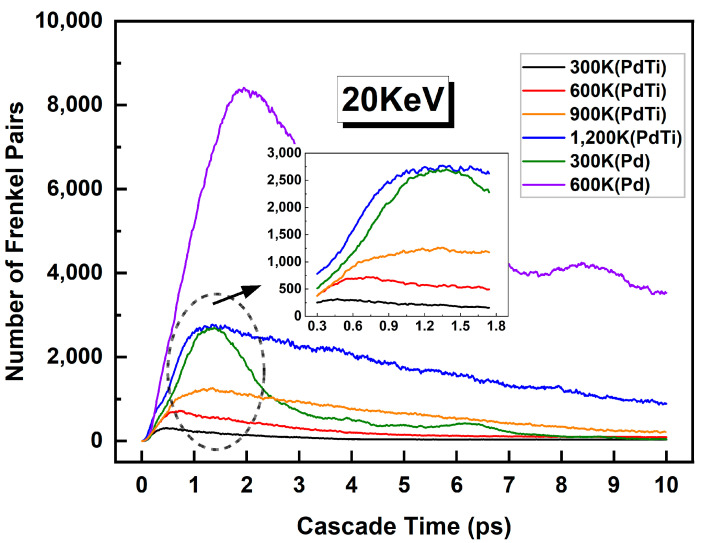
The number of Frenkel pairs as a function of cascade time in Pd and PdTi at different temperatures with a PKA energy of 20 keV. The main plot shows the evolution of the Frenkel pairs over 10 ps, with individual curves representing different temperatures: 300 K, 600 K, 900 K, and 1200 K for PdTi, and 300 K and 600 K for Pd. The arrow points to the inset, which zooms in on the area within the dashed circle, highlighting the peak of the Frenkel pairs.

Figure 12 shows the number of Frenkel pairs as a function of cascade time in Pd and PdTi at different temperatures with a PKA energy of 20 keV. The main plot illustrates the evolution of Frenkel pairs over a period of 10 ps, with each curve representing a different temperature: 300 K, 600 K, 900 K, and 1200 K for PdTi, and 300 K and 600 K for Pd. The inset zooms in on the peak of Frenkel pairs within the initial stage of the cascade. The green and purple lines represent the evolution of Frenkel pairs in Pd at 300 K and 600 K, respectively. From the graph, it is evident that the cascade collision peak and the number of Frenkel pairs after 10 ps of recombination in Pd at 600 K are significantly higher than those in PdTi. This indicates that an increase in temperature has a significantly detrimental effect on the irradiation resistance of Pd. In contrast, PdTi shows lower numbers of Frenkel pairs both at the cascade collision peak and after 10 ps of recombination at 300 K, 600 K, and 900 K. However, when the temperature increases to 1200 K, there is a significant increase in the cascade collision peak and the number of Frenkel pairs after 10 ps of recombination in PdTi [32].

**Figure 13:**
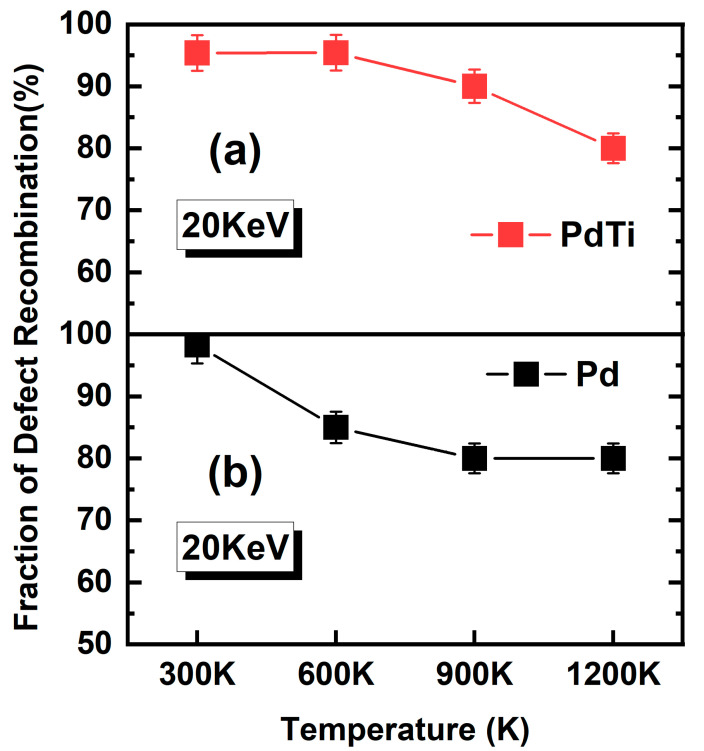
Fraction of defect recombination for PdTi and Pd at different temperatures (300 K, 600 K, 900 K, and 1200 K) with an incident PKA energy of 20 keV. The top panel (**a**) shows the recombination fraction for PdTi, while the bottom panel (**b**) shows the recombination fraction for Pd.

After understanding the images of alloy irradiation at different temperatures, both at the peak of the cascade collisions and after 10 ps of recombination, we proceed to further calculate the defect recombination rate. Figure 13 shows the defect recombination fraction curves for PdTi and Pd at different temperatures (300 K, 600 K, 900 K, and 1200 K) with an incident PKA energy of 20 keV. From the figure, it can be observed that as the temperature increases, the recombination rate for PdTi (a) initially remains high but starts to decrease slightly when the temperature reaches 900 K and continues to decline at 1200 K. In contrast, the recombination rate for Pd (b) shows a significant decline as the temperature rises from 300 K to 1200 K. This indicates that before reaching a certain temperature threshold, the temperature has little effect on the recombination rate of PdTi. However, beyond this threshold, the recombination rate decreases significantly. Compared to pure Pd, PdTi exhibits a significantly higher recombination rate, likely due to the higher entropy of the medium-entropy alloy PdTi compared to pure Pd.

**Figure 14:**
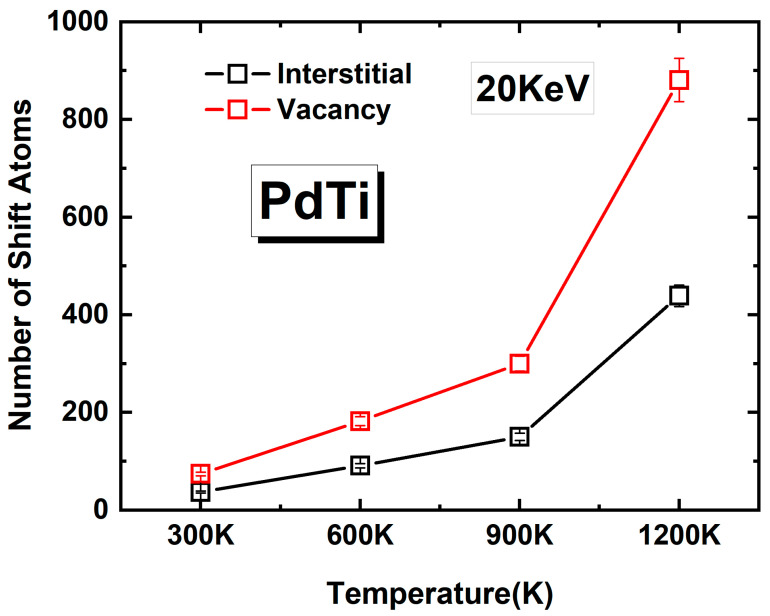
The number of shift atoms in PdTi after irradiation, represented as atoms occupying non-regular lattice sites, at different temperatures (300 K, 600 K, 900 K, and 1200 K) with an incident PKA energy of 20 keV. The graph shows the number of interstitial atoms (black squares) and vacancy atoms (red squares) in the clusters.

Figure 14 shows the number of shift atoms in PdTi after irradiation at different temperatures (300 K, 600 K, 900 K, and 1200 K) with an incident PKA energy of 20 keV. These shift atoms refer to atoms occupying non-regular lattice sites. The graph distinguishes between interstitial atoms (black squares) and vacancy atoms (red squares) in the clusters. From the figure, it is evident that both the numbers of interstitial atoms and vacancy atoms increase with temperature. At 300 K, the numbers of interstitial and vacancy atoms are relatively low. As the temperature increases to 600 K, the numbers of both types of shift atoms increase slightly. At 900 K, the number of vacancy atoms increases significantly compared to interstitial atoms, indicating that higher temperatures favor the formation of vacancies. At 1200 K, there is a sharp increase in the numbers of both interstitial and vacancy atoms, with the increase in vacancies being particularly pronounced. Overall, the data suggest that the effect of temperature on PdTi’s resistance to irradiation damage is primarily due to the increased number of vacancy atoms. While PdTi maintains a relatively stable defect structure at temperatures up to 900 K, at 1200 K, the number of defects, especially vacancies, increases significantly, which could lead to a considerable weakening of PdTi’s irradiation resistance. The data indicate that PdTi may retain good irradiation resistance at temperatures up to 900 K.

**Figure 15:**
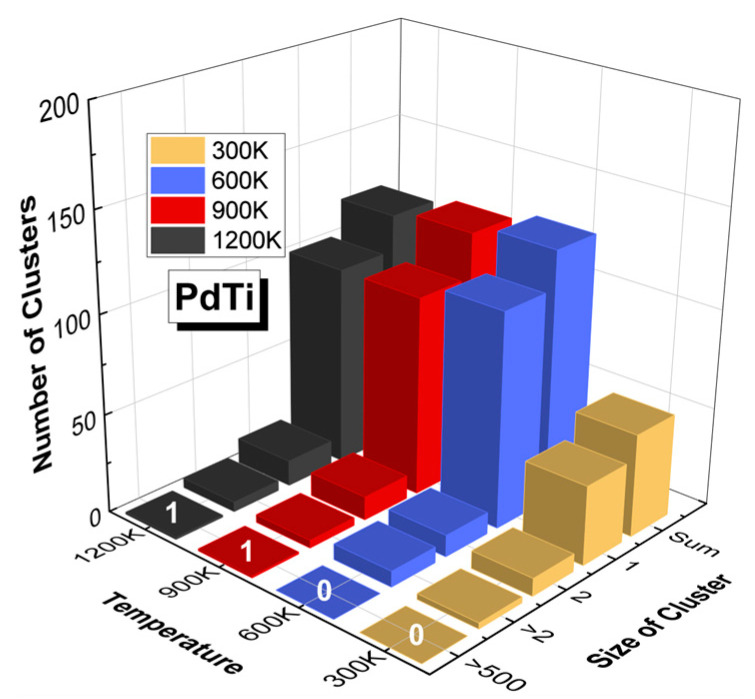
Number of defect clusters in PdTi at different temperatures (300 K, 600 K, 900 K, and 1200 K) with an incident PKA energy of 20 keV, categorized by cluster size. The graph shows the distribution of defect clusters of various sizes formed at different temperatures. The numbers (0, 1) on top of the bars represent the count of clusters with more than 500 atoms in PdTi at different temperatures.

Figure 15 illustrates the number of defect clusters in PdTi at various temperatures (300 K, 600 K, 900 K, and 1200 K) with an incident PKA energy of 20 keV, categorized by cluster size. From this figure, it is evident that an increase in temperature has no significant impact on the size of the clusters. At all temperatures, the defect clusters remain relatively small. Even at the highest temperature of 1200 K, there is no notable increase in cluster size, with the most abundant clusters being those of size 2, totaling 13 clusters. The distribution of cluster sizes remains fairly consistent across the different temperatures, indicating that temperature is not a primary factor affecting the size of defect clusters in PdTi. The formation and size of defect clusters in PdTi may be more significantly influenced by the increase in vacancy atoms resulting from the rise in temperature.

**Figure 16:**
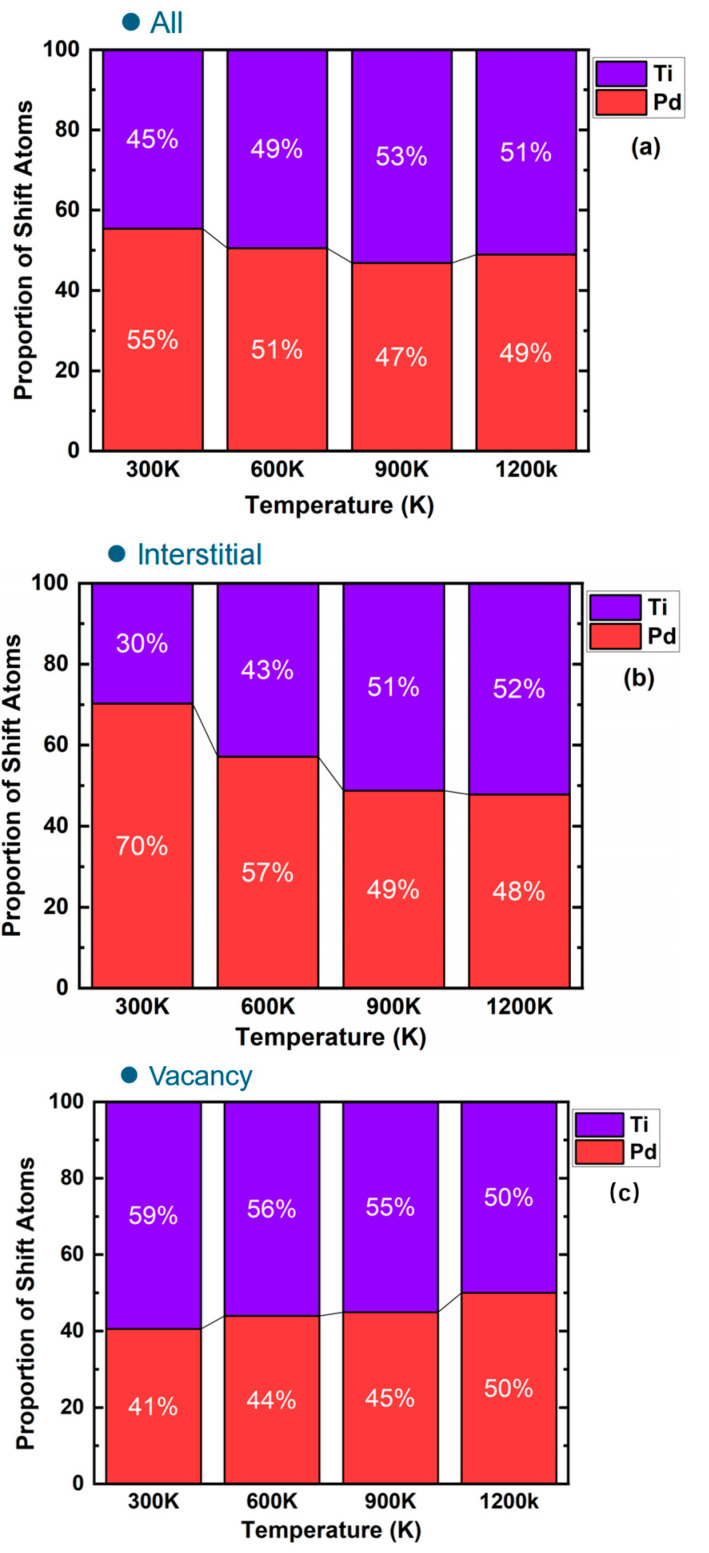
Proportion of Pd and Ti elements in PdTi after 10 ps of recombination at different temperatures (300 K, 600 K, 900 K, and 1200 K): (**a**) proportion of Pd and Ti in clusters of all atoms, (**b**) proportion of Pd and Ti in clusters of interstitial atoms, and (**c**) proportion of Pd and Ti in clusters of vacancy atoms.

Figure 16 shows the proportion of Pd and Ti elements in PdTi after 10 ps of recombination at different temperatures (300 K, 600 K, 900 K, and 1200 K), where (a) shows the proportion of Pd and Ti in clusters composed of all atoms, (b) shows the proportion of Pd and Ti in clusters composed of interstitial atoms, and (c) shows the proportion of Pd and Ti in clusters composed of vacancy atoms. Observing Figure 16a, it is evident that as the temperature increases from 300 K to 600 K and then to 900 K, the proportion of Ti in the clusters gradually rises. However, as the temperature increases from 900 K to 1200 K, the proportion of Pd becomes dominant. The overall trend in Figure 13b, showing the proportion of Pd and Ti in clusters composed of interstitial atoms, is similar to that in Figure 16a for clusters composed of all atoms. From Figure 14, we conclude that the effect of temperature on PdTi’s resistance to irradiation damage is primarily due to the increased number of vacancy atoms. By observing Figure 16c, it is clear that as the temperature rises, the proportion of Pd in the clusters composed of vacancy atoms consistently increases, indicating that higher temperatures may promote the formation of more Pd vacancies.

## 4. Conclusions

This study conducted molecular dynamics simulations to investigate the defect behavior in PdTi medium-entropy alloys under different irradiation conditions. The simulations employed the Large-scale Atomic/Molecular Massively Parallel Simulator (LAMMPS) and utilized the modified embedded-atom method (MEAM) potential to describe the interatomic interactions. Various temperatures, PKA energies, and elemental ratios were tested to understand the formation and evolution of defects.

The results show that compared to pure Pd, PdTi_1.5_, PdTi, and Pd_1.5_Ti alloys, likely due to increased entropy, exhibit superior irradiation resistance at higher temperatures and PKA energies. We investigated different elemental ratios, including Pd, PdTi_1.5_, PdTi, and Pd_1.5_Ti, to explore the impact of element content on the irradiation resistance of non-equimolar PdTi alloys. The findings indicate that varying the elemental ratios influences the defect behavior and irradiation resistance, with irradiation resistance improving as the configurational entropy of the PdTi alloys increases. When the PKA energy increases from 20 keV to 30 keV, the Pd component in the PdTi alloy shows stability, while Ti atoms are more likely to be displaced into interstitial positions due to irradiation, leading to an increase in the number of atoms in defect clusters. 

Defect cluster analysis indicated that before reaching a certain temperature threshold, temperature has little effect on the recombination rate of PdTi, and the size and quantity of defect clusters are not significantly affected by temperature. At temperatures up to 900 K, PdTi exhibited higher recombination rates, suggesting good irradiation resistance. However, as the temperature rises to 1200 K, temperature significantly impacts defect recombination rates, with a continuous increase in both interstitial and vacancy atoms. Specifically, with increasing temperature, more vacancies are formed from Pd atoms, leading to many atoms remaining out of place even after 10 ps of irradiation. This could potentially weaken the alloy’s irradiation resistance. Therefore, the results of this study indicate that more Pd is not always better. Overall, as the environmental temperature of PdTi increases and radiation intensifies, the PdTi alloy with equal proportions of Pd and Ti is likely to exhibit the best irradiation resistance.

For PdTi alloys, the focus is typically on defect behavior and material stability under irradiation rather than photon-induced charge carriers. However, understanding the charge carrier dynamics of these alloys under high-energy conditions may provide insights into their electronic properties and performance in radiation environments. Understanding how electrons are transferred is crucial for explaining complex electronic processes and defect-formation mechanisms. Understanding the behavior of these alloys under photon irradiation (especially in high-energy photon environments) could be important for applications in radiation environments or when considering hybrid materials that combine metal alloys with metal oxides. Moreover, the trade-offs of Pd content in PdTi alloys in terms of mechanical and thermal properties have not yet been thoroughly explored. In future research, a comprehensive evaluation of the material’s irradiation damage resistance, mechanical properties [63], thermal properties, and other aspects [64] to determine the optimal ratio of alloy components will be crucial for accelerating the material’s application.

## Figures and Tables

**Table 1:** PKA atomic parameters.

PKA Atom	Irradiation Energy (KeV)	Velocity (Å/ps)	Number of Experiments
Pd	10	1345.36	5
Pd	20	1897.37	5
Pd	30	2330.24	5

## Data Availability

Please contact the corresponding author for data related to this article.
